# The pig as an amplifying host for new and emerging zoonotic viruses

**DOI:** 10.1016/j.onehlt.2022.100384

**Published:** 2022-04-01

**Authors:** Rebecca K. McLean, Simon P. Graham

**Affiliations:** The Pirbright Institute, Pirbright, Surrey, United Kingdom

**Keywords:** Pig, Amplifying host, Intermediate host, Pandemic, Zoonosis, Viruses

## Abstract

Pig production is a rapidly growing segment of the global livestock sector, especially in Asia and Africa. Expansion and intensification of pig production has resulted in significant changes to traditional pig husbandry practices leading to an environment conducive to increased emergence and spread of infectious diseases. These include a number of zoonotic viruses including influenza, Japanese encephalitis, Nipah and coronaviruses. Pigs are known to independently facilitate the creation of novel reassortant influenza A virus strains, capable of causing pandemics. Moreover, pigs play a role in the amplification of Japanese encephalitis virus, transmitted by mosquito vectors found in areas inhabited by over half the world's human population. Furthermore, pigs acted as an amplifying host in the first and still most severe outbreak of Nipah virus in Malaysia, that necessitated the culling over 1 million pigs. Finally, novel porcine coronaviruses are being discovered in high pig-density countries which have pandemic potential. In this review, we discuss the role that pigs play as intermediate/amplifying hosts for zoonotic viruses with pandemic potential and consider how multivalent vaccination of pigs could in turn safeguard human health.

## List of abbreviations

**Table:** 

CFR	case fatality rate
CoV	coronavirus
CSFV	classical swine fever virus
HA	haemagglutinin
HeV	Hendra virus
IAV	influenza A virus
JE	Japanese encephalitis
JEV	Japanese encephalitis virus
LMIC	low- and middle-income country
MDA	maternally derived antibodies
NiV	Nipah virus
NiV_B_	Bangladesh strain of NiV
NiV_M_	Malaysia strain of NiV
PCV2	porcine circovirus type 2
PEDV	porcine epidemic diarrhoea virus
PRCV	porcine respiratory coronavirus
PRRSV	porcine reproductive and respiratory syndrome virus
SADS-CoV	swine acute diarrhoea syndrome coronavirus
SARS-CoV-2	severe acute respiratory syndrome coronavirus 2
SI	swine influenza
TGEV	transmissible gastroenteritis virus

## 1 Introduction

Within agriculture, the pig sector is the fastest growing livestock subsector alongside poultry [1]. Growth within the pig sector is mostly occurring in low- and middle-income countries (LMICs) with semi-commercial and backyard farmers still accounting for the majority of pig production [2,3]. In some instances, livestock can act as intermediate or amplifying hosts that then leads to disease outbreaks in humans. Viruses that are endemic in pig populations also pose a significant threat to public health. Due to the continuous expanding human population, and the simultaneous increase in the demand for animal protein, people and livestock are now living in closer contact with wild animals, particularly in LMICs [4]. The use of previously unused land has led to an increase in the disruption of wildlife habitats. The increased interaction between humans, livestock and wildlife has led to a rise in virus spillover events from wildlife reservoir species, which in turn has elevated the likelihood of pandemics of new and emerging zoonotic diseases. Furthermore, the international movement of humans as well as animals and animal products has significantly increased, aiding the spread of diseases not only across borders, but worldwide [5]. ‘One Health’ recognises that human, animal, and environmental health are tightly interconnected [6]. One Health is not a new initiative, but it has an ever-increasing importance in the world today.

The development of safe and efficacious vaccines is a strong strategy for the prevention and control of viral outbreaks. Moreover, the vaccination of amplifying/intermediate hosts provides an effective way to further protect human health [7]. A challenge which needs to be addressed for this One Health strategy to be viable for rare diseases or those that may not cause significant disease burden in livestock is the cost of such vaccines. One solution could be the development of bi-or multivalent vaccines to target multiple infectious diseases by a single immunisation. Bi/multivalent vaccines could be produced at little or no extra cost to farmers and complement the vaccines already in place as routine vaccination regimes.

There are a number of zoonotic viruses which can be spread from pigs, including hepatitis E, Ebola and Zika viruses [[8], [9], [10]]. In this review, we discuss the role that pigs play as intermediate and amplifying hosts for three zoonotic viruses with pandemic potential; influenza, Japanese encephalitis and Nipah viruses, as spillover hosts for novel coronaviruses, and how vaccination strategies could help protect the lives and livelihoods of humans.

## 2 Influenza virus: A global pathogen and causative agent of multiple pandemics

Influenza viruses are of worldwide importance, infecting humans, mammalian, and avian species [11,12]. The ability of influenza viruses' segmented genomes to undergo reassortment is a unique trait in which progeny viruses can be produced which have distinctive biological and antigenic characteristics. Specifically, pandemics have previously occurred when an influenza A virus (IAV) with a novel hemagglutinin (HA) surface antigen carries out human-to-human transmission. The ability of IAV to undergo reassortment has caused catastrophic human epidemics and pandemics [12]. The pandemic IAV strains from the past 5 decades have all been reassortants originating from both human and animal strains [12,13]. Pigs are regarded as ‘mixing vessels’ as they are susceptible to avian, swine, and human IAV. In turn, this suggests they are capable of producing novel IAV strains with the potential for pandemic spread [12,14,15]. In 2009, a novel IAV caused an influenza pandemic [16,17]. This virus was the result of triple-reassortant whereby there were six gene segments from at least three parent viruses and two separate segments from the H1N1 swine virus lineage included in the novel IAV [[18], [19], [20]]. This pandemic virus caused a disproportionate amount of disease in the younger population but had a level of virulence much like that of seasonal influenza [17]. The 2009 pandemic H1N1 strain strongly demonstrates the important role of pigs in future outbreaks [12,17,19,21].

Since pigs can independently facilitate the emergence of novel IAV strains with pandemic potential [13,20], continuous monitoring and assessment of emerging influenza viruses in pigs is necessary to avoid future pandemics with devastating effects [12]. In 2020, a novel Eurasian avian-like H1N1 IAV reassortant which possessed genes from the 2009 pandemic and triple-reassortant-strains, termed as the G4 genotype, was isolated following surveillance of pigs in China [12]. This G4 IAV was antigenically distinct from circulating human IAV and possessed all the essential characteristics of a pandemic virus as it preferentially bound to α2–6 sialylated glycans which is a key prerequisite for the infection of human cells and is notably the same binding site as the 2009 pandemic IAV H1N1 strain [12]. Worryingly, the G4 virus showed increased pathogenicity in ferrets and mice [12]. An investigation into the seroprevalence in humans who were unlikely to have contact with pigs, showed a lack of antibodies against the G4 virus, whereas swine-exposed adults showed 10.4% seroprevalence [12]. This work suggests that the G4 virus could be spread from pigs to the human population. If this virus were to evolve and in turn replicate in human cells, the risk of a pandemic is increased significantly. Despite this inherent risk, and lessons which should have been learnt from past experiences, the risk of another influenza pandemic remains high.

Swine influenza (SI) is a key player in the multifactorial porcine respiratory disease complex. Globally, H1 and H3 subtypes circulate in pigs with various combinations of N1 and N2 subtypes; however, strains vary greatly by continent. The continuous, rapid evolution of SI continues to be a major obstacle despite immunisation being a cost-effective control measure [22,23]. Importantly, not all SI-endemic countries use vaccines to control disease. In the UK, the current policy does not involve immunisation against SI, although it is used widely in the USA and in some European countries [22]. Furthermore, inactivated vaccines have been shown to induce low levels of cross-reactivity between strains, which is concerning. This is thought to be due to antigenic drift of the surface glycoproteins causing rapid viral escape [24,25]. A further challenge with the vaccination of pigs occurs due to the circulation of several virus lineages at the same time in the same location. For a successful vaccination regime here, broadly cross-reacting antigens would be required [25]. Due to these complications, novel strategies and platforms for vaccination are needed to safeguard human and animal health.

## 3 Japanese encephalitis virus: a common cause of encephalitis in Asia

Japanese encephalitis virus (JEV) is an enveloped, positive sense, single stranded RNA virus within the Flavivirus genus of the *Flaviviridae* family [26]. JEV is closely related to dengue, yellow fever and West Nile viruses [27]. Twenty-six countries in Southeast Asia and Western Pacific regions have a JEV transmission risk, which encompasses over half of the global human population [26]. *Culex* spp. mosquitoes transmit JEV from animals to humans [[28], [29], [30]]. Ardeid birds are thought to be the natural reservoirs for JEV, pigs in turn are the main amplifying host, and *Culex* mosquitoes are the vectors in the transmission cycle [[30], [31], [32]]. Interestingly, more recent findings have implicated that direct transmission between pigs can occur via oronasal virus shedding [33]. There are several factors which favour the pig as the main amplifying host for JEV, such as the constant production of immunologically naïve piglets [33]. Furthermore, an important vector for JEV, *C. tritaeniorhynchus*, preferentially feed on pigs over other animals [31].

Safe and effective JE vaccines for use in humans have been shown to control disease in endemic countries [34]. There are currently four main JE vaccines licensed for use, which are either derived from inactivated mouse brain or inactivated Vero cells; live attenuated vaccines, or live recombinant chimeric vaccines [27]. The most commonly used vaccine is the SA 14–14-2 live attenuated JEV vaccine, which has been used throughout China for more than two decades, providing up to 80% protection from disease after a single dose, which increases to 97.5% after a prime-boost schedule within 1-year [35,36]. Furthermore, a single dose was 99.3% effective in preventing clinical disease in Nepal [36,37] with protection marginally lessening to 98.5% after 15 months [38]. Despite these successes, concerns surrounding the price, supply, adverse effects, and efficacy have made it challenging for LMICs to control the disease. Inactivated JE vaccines derived from mouse brain have previously been used in India, but the production capacity was insufficient to meet the national need [39,40].

Clinical signs in pigs naturally infected with JEV are generally mild but have been associated with increased reproductive issues [41], with viremia lasting approximately 2–4 days [32]. High viremia in pigs could lead to an increase in the spread to humans and therefore another potential strategy in which to control JE could be the vaccination of pigs. There have been a number of vaccine candidates assessed for protective efficacy in pigs including live-attenuated [42]; lentiviral vectored [43,44] and DNA vaccines [45]. Each of these vaccines produced immune responses in pigs great enough to stop onward transmission by mosquitoes. Even more impressive was that one lentiviral vector vaccine showed protection against three different JEV genotypes [44]. These data suggest a One Health approach to vaccinating pigs could be beneficial in countries where JEV is prevalent to help control the rates of infection. However, this is not widely used for a number of reasons. Firstly, it is not thought to be economical due to the high turnover in pig populations, requiring farmers to vaccinate frequently. Secondly, the use of live attenuated vaccines in young pigs is questionable as their effectiveness is reduced due to maternally derived antibodies (MDA) [26]. However, using viral or DNA vectored vaccines would counteract this issue as they are able to overcome MDA interference [46].

## 4 Nipah virus: a blast from the past with emerging pandemic potential

Nipah virus (NiV) is an enveloped, single stranded, negative sense RNA paramyxovirus [7]. NiV and the related Hendra virus (HeV) both have a broad host tropism due to the use of the conserved Ephrin B2 and B3 as their cellular receptors [7,47,48]. Furthermore, NiV and HeV are generally larger than other paramyxoviruses and show high levels of cross-reactivity on serological tests. This led NiV and HeV to be grouped together in the *Henipavirus* genus [49]. Two distinct strains of NiV have been well characterised, Malaysia (NiV_M_) and Bangladesh (NiV_B_). The strains are 91.8% identical at a nucleic acid level but differ in their pathogenicity and transmissibility [50,51]. For example, NiV_M_ was the causative agent behind the Malaysian outbreak, and had an approximate case fatality rate (CFR) of 40%. NiV_B_ on the other hand, has caused repeated outbreaks since 2001 in Bangladesh and has an average CFR of 75%. There is also evidence for human-to-human transmission of NiV_B_, which was not seen during the NiV_M_ outbreak [[51], [52], [53], [54]].

The first and most devastating outbreak of NiV occurred in the Perak state in Peninsular Malaysia with the first virus isolate identified in an encephalitic patient from the Sungai Nipah village in 1999 [55]. This outbreak of encephalitis in humans followed an outbreak of respiratory illness and encephalitis in pigs from within the same district [56]; however, at the time, these two scenarios were not thought to be linked. As the role of pigs was not understood at this point, NiV was spread by the movement of infected pigs throughout Malaysia and into Singapore [57]. However, the link to pigs became clear as 93% of the infected humans had direct contact with pigs [7,58]. It is widely documented that *Pteropus* spp. bats, which roosted near pig barns, transmitted the virus to the pigs which in turn amplified the virus and passed it onto humans. The Malaysian Ministry of Health carried out disease surveillance for over a year (29th September 1998 to December 1999), reporting 283 cases of viral encephalitis, of which there were 109 fatalities [55]. The outbreak in Malaysia and Singapore was halted by the culling of 45% of the national pig herd [58,59]. This outbreak caused significant economic costs to the sum of US$ 582 million; which included US$ 97 million of compensation to cover the slaughter of 1.1 million pigs and the loss of 36,000 jobs within the pig farming sector in Malaysia [7]. Singapore prohibited the import of live pigs from 1999 to 2017 because of this outbreak.

In May 2018, the first outbreak of NiV in Kerala, southern India, was described [60]. In total there were 19 NiV cases, of which 17 resulted in fatalities. *Pteropus giganteus* bats from areas around Kozhikode, Kerala, were assessed for NiV at the National High Security Animal Diseases Laboratory at Bhopal whereby 19% were found to be NiV positive by RT-PCR [7,61]. It is thought that NiV has a high pandemic potential as it can spillover from intermediate animal hosts to humans and cause outbreaks of person-to-person transmission [7,62].

There are several characteristics that predispose NiV to becoming a global pandemic. These include: human susceptibility; person-to-person transmission; and has a high mutation rate [63]. Humanity could face a devastating pandemic if a human-adapted NiV strain were to infect communities in Southeast Asia where there are high human and pig densities and pigs are a primary export good [62,64,65]. Due to the significant role pigs played in the first and most severe outbreak of NiV, a One Health approach whereby pigs are vaccinated to prevent the spread of the virus, either routinely or in an outbreak situation, should be considered. Previous work assessed the protective efficacy of an adjuvanted HeV soluble envelope glycoprotein (sG) subunit vaccine (Equivac® HeV, Zoetis) in pigs. This vaccine is licensed to protect horses against HeV in Australia, as well as reducing the zoonotic risk to humans of infection. Despite Equivac® HeV protecting both ferrets and African green monkeys (AGMs) from NiV infection after experimental challenge, the vaccine failed to protect pigs [7,[66], [67], [68]]. It was concluded from these studies that protection requires both humoral and cellular immune responses as the pigs did not mount a measurable T cell response [68]. Conversely, a canarypox virus (ALVAC strain) vector expressing NiV G or fusion (F) proteins (ALVAC-G and ALVAC-F) have been found to protect pigs against virulent NiV challenge after a prime-boost regime [69]. Most recently, we evaluated the immunogenicity of recombinant bovine herpesvirus-4 (BoHV-4) vectors expressing either NiV G or NiV F against ALVAC-G in pigs. Both BoHV-4 vectors induced potent antibody and T cell responses in comparison to ALVAC-G but as no challenge was performed, protective efficacy was not determined [70].

Despite these promising results, no vaccine has progressed toward licensing for either pigs or humans. Nevertheless, the encouraging performance of NiV vaccine candidates in large animal models thus far firmly supports the idea that a safe and efficacious vaccination against NiV can be developed for pigs which will in turn reduce the chance of a future outbreak and reduce the continued public health threat. Accordingly, we are currently evaluating the protective efficacy of three novel NiV vaccine candidates in pigs with the view to developing a prototype vaccine for further development [7].

## 5 Coronaviruses have a history of mutating to gain advantage in the host and to jump host species

Coronaviruses (CoV) are enveloped viruses with large positive-sense, single-stranded RNA genomes [71]. There are thought to be hundreds of coronavirus species circulating in a wide range of mammalian species including pigs, camels, bats, and humans. Seven CoV are known to infect humans causing disease. HCoV-OC43, HCoV-229E, HKU1, and HCoV-NL63 cause mild to moderate disease [72]. However, three can cause potentially fatal disease: SARS coronavirus (SARS-CoV), the causative agent of severe acute respiratory syndrome (SARS) [73]; Middle East respiratory syndrome coronavirus (MERS-CoV), which has continually caused sporadic, localised outbreaks since 2012 [74], and can be transmitted from infected dromedary camels [75]; and finally SARS-CoV-2, which emerged from China in late 2019 and is the causative agent of coronavirus disease 2019 (COVID-19). COVID-19 was declared a global pandemic by the World Health Organization on March 11, 2020 [76,77]. As it is now well documented that CoV can spillover from animals into humans and cause fatal disease, it is vital to monitor potential zoonotic viruses in wildlife and livestock reservoirs for the development of advantageous mutations.

An interesting example of a CoV mutating to gain advantage is the transmissible gastroenteritis virus (TGEV) which is highly virulent, causing nearly 100% mortality in neonatal piglets. TGEV mutated to produce a truncated spike protein [78] that altered the tropism to the respiratory tract from the gastrointestinal tract thus creating a new virus - porcine respiratory coronavirus (PRCV) [79,80]. The worldwide decline of TGEV has been widely attributed to cross-immunity following PRCV infection due to cross-reactive neutralising antibodies [81]. Instead, PRCV has become endemic and highly prevalent in most countries where pigs are bred [82].

In the early 1970s, the CoV referred to as porcine epidemic diarrhoea virus (PEDV), swept through Europe. Outbreaks of PEDV have been reported in Asia and North America since the initial spread in Europe [83]. Interestingly, from the 1980s onwards PEDV outbreaks became infrequent in Europe. The reason for this apparent resistance to disease remains unknown [83,84]. In 2010, new variants of PEDV were reported in China. PEDV outbreaks were seen in vaccinated pigs which questioned the effectiveness of vaccines, which had previously been successfully used to control the virus since the 1990s [83,85]. In early 2013, PEDV arrived in North America which resulted in huge economic losses [86] and by late 2013, PEDV outbreaks occurred in Japan, Taiwan and South Korea, where prevalence had previously been low. Phylogenetic analysis highlighted that the PEDV strain isolated from Taiwan were in the same clade as the strains circulating in the US [87]. PEDV is now a globally recognised re-emerging pathogen which is causing major financial issues worldwide.

Most recently, swine acute diarrhoea syndrome coronavirus (SADS-CoV) was discovered in the Guangdong province of China, 2016 [88]. SADS-CoV is a highly pathogenic virus which is thought to have evolved from the closely related bat coronavirus HKU2 [89]. SADS-CoV caused a major fatal disease outbreak on four farms totalling 24,693 piglet deaths, which interestingly began 100 km from the origin of the SARS pandemic [88]. Humans are potentially susceptible to SADS-CoV infection as previous work has shown the virus infects and grows in human primary cells derived from both the lung and intestine [89,90].

Due to the convoluted history of porcine coronaviruses, and their propensity for rapid and radical mutations altering tropism as well as pathogenicity, there is an inherent risk of pigs producing (or amplifying) a novel strain of coronavirus capable of infecting humans. Currently the prevention and control of porcine CoV relies on high biosecurity measures and disease containment rather than vaccination programmes (PEDV being the exception) [91]. However, coronaviruses frequently spillover to other species [91,92] meaning there is a potential for the emergence of novel human and animal coronaviruses, thus demanding novel approaches to characterise the potential threat of coronaviruses to human and animal health.

## 6 Immunising pigs with bi/multivalent vaccines could help prevent zoonotic disease epidemics

Vaccination of intermediate porcine hosts could be an important tool to help protect humans from viruses with pandemic potential [93]. However, a significant challenge to achieving this is the lack of a viable market. Historically, there has been an understandable hesitancy from farmers to pay for vaccines which will not directly improve the productivity of their livestock. This is true both of large scale and small holder farmers. Therefore, if vaccines could be produced which complement and do not interfere with existing vaccination regimes, pig farmers would be more likely to take up this practice. This could be implemented using bi- or multi-valent vaccines. Multivalent vaccination is a strategy the animal health industry is already moving toward and an active research area. One example is the use of live attenuated porcine reproductive and respiratory syndrome virus (PRRSV (*Betaarterivirus suid*)) as a modified viral vector to express other viral antigens [[94], [95], [96]]. PRRSV has been evaluated experimentally as a vector for delivery of the capsid antigen from porcine circovirus type 2 and haemagglutinin from swine IAV. Currently, each of these three diseases are vaccinated against individually, which is costly. This tri-valent vaccine was shown to confer protection against all three viruses [95], although the genetic stability of PRRSV vectors needs to be improved [95,97]. Nevertheless, this shows potential for the use of PRRSV as a viral vector. Another candidate vector is live attenuated pseudorabies virus (Suid herpes virus 1; SuHV-1). Pseudorabies remains endemic in Asia and live attenuated vaccines are widely used to control this disease. These vaccines are highly effective, playing a major role in the eradication of SuHV-1 from Western Europe and North America [98]. With a large genome and several non-essential genes, SuHV-1 has the capacity to harbour exogenous genes, and therefore the ability to express heterologous antigens [99]. Recombinant live attenuated pseudorabies virus expressing PRRSV antigens are both immunogenic and efficacious [100,101]. Furthermore, recombinant pseudorabies virus vectors have been shown to protect piglets from classical swine fever virus (CSFV) after lethal experimental challenge when immunised with a pseudorabies vector expressing the E2 gene from CSFV [[102], [103], [104]].

A bi/multivalent approach could therefore be explored to include antigens from zoonotic viruses, such as JEV and NiV. If successful, such vaccines could be routinely used to immunise pig herds against PRRS or pseudorabies, and which, at no/minimal additional cost to the farmers, would also reduce the risk of pigs acting as amplifying hosts and thus protect both their livelihoods and public health. Despite the previous positive results shown using bivalent vaccines in pigs, none are currently used in the field. We are working with colleagues to test this concept, initially in the context of NiV, and are engineering live attenuated PRRSV and SuHV-1 vectors expressing the sG or sF antigens.

## 7 Concluding remarks

Pigs can act as intermediate and amplifying hosts for viruses with pandemic potential including NiV, IAV and JEV, and they have become established as hosts for novel coronaviruses. Despite the acceptance of their role in the ecology of these diseases, in many instances, little has been done to prevent recurrences. A One Health approach, where pigs are routinely vaccinated in order to prevent future pandemics and safeguard human health is vital. The use of routine vaccination in livestock for zoonotic diseases, potentially via recombinant bi/multivalent vaccines, as well as increased active surveillance for viruses circulating in pig herds could both be used to reduce the pandemic risk these pathogens pose.

## Ethical Approval and Consent to participate

Not applicable.

## Consent for publication

Not applicable.

## Availability of supporting data

Not applicable.

## Funding

We gratefully acknowledge financial support from the UK Department for Health and Social Care (SBRI Vaccines for Global Epidemics—Clinical Contract−971555 A Nipah vaccine to eliminate porcine reservoirs and safeguard human health) and The 10.13039/501100000870Pirbright Institute Internal Seed Fund. SPG is supported by a 10.13039/501100000268UKRI Biotechnology and Biological Sciences Research Council (BBSRC) Institute Strategic Programme Grant to the 10.13039/501100000870Pirbright Institute (BBS/E/I/00007031).

## Authors' contributions

Manuscript written by RKM and SPG.

## Declaration of Competing Interest

None to declare.
